# Emotional response of critically-ill cardiac patients during hygiene procedures in intensive care: a prospective and descriptive study

**DOI:** 10.1590/1518-8345.6808.4032

**Published:** 2023-11-03

**Authors:** Silvia Pérez-Ortega, Elena Querol Vallés, Judith Prats Barrera, Montserrat Venturas, Adelaida Zabalegui

**Affiliations:** 1 Hospital Clínic Barcelona, Barcelona, España.; 2 University of Barcelona, Barcelona, España.

**Keywords:** Hygiene, Skin Care, Critical Care, Nurses, Emotions, Coronary Care Units, Higiene, Cuidados de la Piel, Cuidados Críticos, Enfermeras y Enfermeros, Emociones, Unidades de Cuidados Coronarios, Higiene, Higiene da Pele, Cuidados Críticos, Enfermeiras e Enfermeiros, Emoções, Unidades de Cuidados Coronarianos

## Abstract

**Objective:**

to analyze the emotional response of critically-ill conscious patients during daily hygiene procedures in a Cardiology Intensive Care Unit and to compare it based on the existence of previous experiences or not.

**Method:**

a prospective and descriptive study. A 30-item ad hoc survey based on the first-day hygiene procedures was applied to 148 patients. Questions are asked about the feelings during the hygiene procedures and about positive and negative aspects of the experience. The patients are compared based on whether they had been already subjected to hygiene procedures or not.

**Results:**

67.6% were men and their mean age was 67±15 years old; 45.9% proved to be satisfied, 27% felt embarrassment and 86.3% were grateful to the professionals for talking to them during the hygiene procedures. 33.1% of the patients had never been subjected to hygiene procedures in bed, were significantly younger and single, and presented a lower cleanliness sensation. 32% stated that they would like for a family member to collaborate in the hygiene procedures.

**Conclusion:**

the patients do not feel that their intimacy is invaded when they are subjected to hygiene procedures and appreciate communication with the health personnel while this care is provided. Those who had never been subjected to hygiene procedures in bed are younger, feel more embarrassed and are more disturbed by interruptions, in addition to being more aware of them.

## Abstract (es)

**Objetivo:**

analizar la respuesta emocional de pacientes conscientes en estado crítico durante la higiene diaria en una unidad de cuidados intensivos cardiológicos y compararla en función de experiencias previas o no.

**Método:**

estudio prospectivo y descriptivo. Encuesta *ad hoc* de 30 ítems realizada a 148 pacientes y basada en la higiene del primer día. Se formulan preguntas sobre los sentimientos durante la higiene y aspectos positivos y negativos de la experiencia. Se compara a los pacientes en función de si se los había higienizado con anterioridad.

**Resultados:**

el 67,6% fueron hombres y la media de edad fue de 67±15 años. El 45,9% presentó conformismo, el 27% sintió vergüenza y el 86,3% agradeció que le hablaran durante la higiene. Al 33,1% de los pacientes nunca les habían realizado higiene en la cama, eran significativamente más jóvenes y solteros, y tenían menor sensación de limpieza. El 32% expresó que le gustaría que un familiar colaborase en la higiene.

**Conclusión:**

los pacientes no sienten que se invade su intimidad cuando se los higieniza y aprecian la comunicación con el personal sanitario durante estos cuidados. Los pacientes a quienes no les habían realizado higiene en la cama previamente son más jóvenes, sienten mayor vergüenza y les molestan más las interrupciones, siendo más conscientes de ellas.

## Abstract (pt)

**Objetivo:**

analisar a resposta emocional de pacientes críticos conscientes durante a higiene diária em uma unidade de terapia intensiva cardíaca e compará-la considerando ou não as experiências prévias.

**Método:**

estudo prospectivo e descritivo. Foi aplicado questionário ad hoc de 30 itens a 148 pacientes, com base na higiene do primeiro dia. Foram feitas perguntas sobre sentimentos durante a higiene e aspectos positivos e negativos da experiência. Os pacientes foram comparados considerando o fato de terem sido higienizados anteriormente.

**Resultados:**

67,6% eram homens e a idade média foi de 67±15 anos. 45,9% apresentavam conformismo, 27% se sentiram envergonhados e 86,3% estavam gratos por terem conversado com eles durante a higiene; 33,1% dos pacientes acamados nunca haviam recebido cuidados de higiene no leito, eram significativamente mais jovens e solteiros, e tinham um senso de limpeza mais baixo; 32% expressaram que gostariam que um membro da família ajudasse na higiene.

**Conclusão:**

os pacientes não se sentiram invadidos em sua intimidade quando receberam os cuidados de higiene e apreciaram a comunicação com o pessoal de saúde durante o procedimento. Os pacientes que não tinham recebido cuidados de higiene no leito anteriormente são mais jovens, sentem-se mais constrangidos e mais incomodados pelas interrupções, sendo mais conscientes delas.

**Highlights:**

**(1)** It is essential to understand the patients’ emotions for good quality ICU care.

**(2)** An expanded view of what most patients think about hygiene is contributed.

**(3)** The study allows deepening on more intimate aspects of the care provided.

**(4)** The study allows adapting care to the needs stated.

## Introduction

During the last few years, Cardiology Intensive Care Units (CICUs) have evolved into high-complexity units where not only patients with acute ischemic heart disease are treated. Medical and technological advances (ventricular assistance devices, resynchronization therapies and optimization of pharmacological therapies, among others) open new therapeutic windows for patients with acute heart failure or fatal arrhythmias ^(^ 1 ^-^ 3 ^)^ . This implies an increase in survival of these patients and chronification of pathologies with previous fateful prognoses. On the other hand, the increase in the number of therapeutic techniques available, such as percutaneous treatments for valvulopathies or cardiac structural pathology, offer therapeutic options to patients with high comorbidity levels that oftentimes require admission to these units; either as immediate postoperative treatment or in case of any type of complication ^(^ 2 ^)^ . 

It is for this reason that, currently, the individuals that are admitted to a CICU are very complex patients and, due to their criticality, require a series of Nursing care measures that ensure their safety on the one hand and, on the other, technical and perceived assistance quality at all times. The objective of all health providers is to offer patient-centered care seeking to improve the perceived quality of the assistance provided and to obtain better health results ^(^ 4 ^-^ 9 ^)^ . 

Skin and mucosa hygiene and care are part of the everyday activities that Nursing workers should perform, generally in charge of two professionals, in order to help these patients meet their self-care deficit, with the possibility of generating feelings of dependence and vulnerability ^(^ 10 ^-^ 13 ^)^ . 

One of the rights of patients admitted to Intensive Care Units (ICUs) is to be offered individualized and respectful care by physicians and nurses ^(^ 14 ^)^ . Oftentimes, activities such as patients’ hygiene in bed can be routinely performed by the Nursing personnel and produce negative emotions in the patients ^(^ 5 ^,^ 15 ^-^ 17 ^)^ . Some patients are conscious in CICUs; for them, losing their autonomy while performing the hygiene procedures might suppose an invasion to their intimacy. 

Various studies assess anxiety and depression in critically-ill patients, as well as the different stressors that can be manifested in them ^(^ 16 ^,^ 18 ^)^ . However, there is no validated questionnaires to quantify so concrete Nursing care aspects such as the sensations generated by daily hygiene. The feelings and needs of conscious patients during the hygiene procedures performed by the health personnel in bed are unknown. 

As a hypothesis, our study proposes that in ICUs, increasingly technified, we fail to sufficiently take into account the patients’ emotional state when providing this basic care. Knowing the patients’ experience will allow us to define improvement lines in the care we offer, particularly in applying so sensitive and necessary care measures, not only to favor a well-being sensation but also to prevent contagion of healthcare-associated infections.

The study objective is to analyze the emotional response of critically-ill conscious patients during daily hygiene in a Cardiology Intensive Care Unit and to compare it based on the existence of previous experiences or not.

## Method

### Study design

A prospective and descriptive study that followed the guidelines proposed in the Strengthening the Reporting of Observational Studies in Epidemiology (STROBE) checklist ^(^ 19 ^)^ . 

### Locus

Cardiology Intensive Care Unit of Barcelona Clínic Hospital, a high-technology tertiary-level institution.

The CICU in this center has 16 beds. The unit was fully refurbished in 2015 and each patient has their own room, with no bath, and opacifying glass doors that open in full from the outside.

The profile of the patients treated is extremely diverse. Some of them are sedated, on mechanical ventilation, hemodynamically unstable and with invasive circulation support devices; on the other hand, others are conscious at all moments and, although more stable, need to admitted to the CICU due to their high risk of fatal complications.

### Period

Data collection was initiated on July 2021 among all the patients that consecutively met the inclusion criteria and none of the exclusion ones, until reaching the sample size in December 2021.

### Population

An anonymous survey is applied to the patients admitted to the CICU of a high-technology hospital, 24-48 hours after hospitalization and once the hygiene procedure in bed has been performed. The patients considered to take part in the study are those that meet all the inclusion criteria and none of the exclusion ones. The study population corresponded to all patients aged over 18 years old, conscious and admitted to the CICU of a high-technology hospital for a minimum of 48 hours, at rest, already subjected to the hygiene procedure in bed and who agreed to answer the anonymous survey. The patients excluded were those with cognitive decline or unable to answer the survey, as well as those in a clinical situation that precluded answering the survey in the first 48 hours and those that were admitted to the CICU from another unit or from other hospitals, with hospitalization times at origin over 24 hours.

### Definition of the sample

A total of 625 patients were admitted to the CICU during 2018, of which 386 were conscious and, therefore, represented possible candidates to answer the survey. Adjusting losses to 10%, the resulting sample calculation was 148 patients.

### Study variables

The study objective is analyzed through an *ad hoc* questionnaire. This instrument is based on the first-day hygiene procedure. 

A bibliographic review is conducted where no questionnaire is found that assesses different aspects of hygiene in critically-ill patients; however, different aspects that exert an influence on the patients’ health care and well-being perceptions in the ICU are determined, namely: communication with the patient; humanizing interventions; family participation in the care provided to critically-ill patients; sleep deprivation; and pain ^(^ 8 ^,^ 18 ^,^ 20 ^-^ 24 ^)^ . Based on these items, a survey was worked on to assess the patients’ perception and emotions regarding hygiene in bed. An initial test was carried out with 5 individuals to ensure that the questions were clear, simple and understandable for the users. 

In this initial test, 60% were men and their mean age was 70±14 years old. 60% of the patients had never been subjected to hygiene procedures in bed. Of them, 66% stated feeling embarrassed during the hygiene procedures and perceiving that their intimacy was extremely or quite invaded; 40% of the patients were a little or quite upset by interruptions. In 80% of the cases, the hygiene procedures were in charge of two women; however, we did not ask if they patients liked this or not. One patient indicated that he was grateful for being allowed to wash his genitals by himself. Only 1 out of 5 patients stated that they would like for a family member to collaborate in the hygiene procedures.

The initial questionnaire did not reflect some aspects that are relevant for the study, such as whether the patients were comfortable with the professionals during the hygiene procedures or some of their preferences. It is for this reason that, from the first version of the questionnaire to its final one after the test, questions about situations that take place during the hygiene procedures were added, such as communication between professionals and patients; in addition, the preference in terms of number and gender of the professionals collaborating in the technique, as well as in hygiene of the genitals, was also incorporated.

The questionnaire has 30 items [9 multiple-choice questions, 8 that are scored from 1 to 4 in a Likert scale (estimation questions), 12 dichotomous questions and 1 open question]. The questions refer to how each patient felt when subjected to the hygiene procedures, how they were performed, by whom, if they met their preferences, and if they would like for a family member to be involved in this care. Finally, a global assessment is requested and the option to include an open comment is offered.

The main sociodemographic data are also collected (gender, age, marital status, family members living in the same house), diagnosis at admission, and number and duration of the family visits.

### Data collection

The questionnaire is handed in to the conscious patients between 24 and 48 hours after admission to the CICU, once the hygiene procedure in bed has been performed.

The questionnaires are in charge of one of the researchers, who has no direct care dealings with the patients, to avoid conformity bias.

Procedure: following the hospital’s protocol and the recommendations for infection prevention in critically-ill patients ^(^ 11 ^)^ , in this unit the hygiene procedures for these patients have been performed since the beginning of 2021 with chlorhexidine towels, sponges with no soap for the face and soap and water or chlorhexidine towels for the genitals, according to each patient’s needs. The towels are kept at a temperature of 45ºC by means of a heater provided by the company. 

### Data treatment and analysis

The quantitative variables are expressed as mean ± Standard Deviation (SD) or as median and Interquartile Range (Q1-Q3) when not following normality criteria. They are compared by means of the Student’s t or Mann-Whitney’s U tests. The qualitative variables are expressed as percentages and analyzed using the chi-square test. The participants are analyzed based on whether it is the first time that they are subjected to hygiene procedures in bed or not, comparing these two subgroups. Group A included those who had already been subjected to hygiene procedures in bed and Group B was comprised by those who had never undergone these procedures. Significance is considered when p<0.05. The statistical analysis is performed in IBM SPSS v23 software.

### Ethical aspects

The participants were informed about the study, written information was provided and their informed consent was requested.

The study was approved by the Center’s Medication Ethics Committee (HCB/2021/0552).

## Results

All 148 patients that met the inclusion criteria and none of the exclusion ones agreed to take part in the study. Their mean age was 67±15 years old and 67.6% were men; 58.5% were married or had a partner. The patients live with a mean of 2.31±0.9 family members in the same household and 74.7% of them received visits at admission. The main diagnosis at admission was Acute Coronary Syndrome (ACS) with or without ST-segment elevation. Table 1 shows the sociodemographic and clinical characteristics of the study patients. 

**Table 1 -:** Sociodemographic and clinical characteristics of the patients (n=148). Barcelona, Spain, 2021

Variables	
Age (SD ^*^)	67±15
Gender (Female) n (%)	48 (32.4)
Marital status n (%)	
Married	74 (50.3)
With a partner	12 (8.2)
Divorced	16 (10.9)
Single	24 (16.3)
Widowed	21 (14.3)
Number of people in the household (including the patient) (SD ^*^)	2.31±0.9
Visits at admission n (%)	109 (74.4)
Number of visits at admission (SD ^*^)	1.34±0.71
Diagnosis n (%)	
STEMI-ACS ^†^	32 (21.6)
NSTEMI-ACS ^‡^	35 (23.6)
AHF ^§^	5 (3.4)
TAVI ^||^	15 (10.1)
Mitraclip	5 (3.4)
CAVB ^¶^	11 (7.4)
Pericardial effusion	4 (2.7)
Arrhythmic storm	6 (4.1)
Others	35 (23.6)

^*^SD = Standard Deviation; ^†^STEMI-ACS = Acute Myocardial Infarction with ST-segment Elevation; ^‡^NSTEMI-ACS = Acute Myocardial Infarction without ST-segment Elevation; ^§^AHF = Acute Heart Failure; ^||^TAVI = Transcatheter Aortic Valve Implant; ^¶^CAVB = Complete Atrioventricular Block

66.9% of the patients have already been subjected to hygiene procedures in bed during previous hospitalizations. It was significant (p<0.005) that the patients who had never been subjected to such procedures were younger: 54±14 *vs* 73±11 years old. 

Most of the patients were married or had a partner. In fact, it was significant that there were more single individuals among the patients who had never been subjected to hygiene procedures in bed: 9.1% *vs* 31.6%. 

The patients that had never been subjected to hygiene procedures in bed (Group B) felt significantly more embarrassed. On the other hand, those who had already undergone such procedures (Group A) presented more conformity ( Table 2). 

**Table 2 -:** Behavioral aspects: differences based on previous experiences related to hygiene in bed. Barcelona, Spain, 2021

Behavioral aspects	TOTAL (n=148)	Group A Hygiene in bed already performed (n=99)	Group B Hygiene in bed never performed (n=49)	p ^*^
What did you think when they told you that you were going to be subjected to hygiene procedures in bed? n (%)				
Embarrassment	40 (27)	10 (10.1)	30 (61.2)	<0.05
Relief	23 (15.5)	17 (17.2)	6 (12.2)	
Conformity	68 (45.9)	55 (55.6)	13 (26.5)	
Others	17 (11.5)	17 (17.2)	0	
Did you feel that your intimacy was invaded? n (%)				
Absolutely not	86 (58.1)	75 (75.8)	11 (22.4)	<0.05
A little	39 (26.4)	21 (21.2)	18 (36.7)	
Pretty much	12 (8.1)	2 (2)	10 (20.4)	
A lot	11 (7.4)	1 (1)	10 (20.4)	
How many people performed the hygiene procedures? (SD ^†^)	1.96±0.2	1.97±0.17	1.94±0.24	NS ^‡^
How many people would you have preferred? n (%)				
The same	134 (90.5)	97 (98)	37 (75.5)	<0.05
Fewer	14 (9.5)	2 (2)	12 (24.5)	
Did you like being talked to during the hygiene procedures?				
n (%) YES	119 (82.6)	83 (87.4)	36 (73.5)	0.037
Did you like being told stories during the hygiene procedures?				
n (%) YES	107 (74.3)	75 (78.9)	32 (65.3)	NS ^‡^
Did you like their professionalism?				
n (%) YES	110 (76.9)	76 (80.9)	34 (69.4)	NS ^‡^
Did you like the cleanliness sensation?				
n (%) YES	96 (66.7)	69 (72.6)	27 (55.1)	0.028
How did they perform the hygiene procedures? n (%)				
Chlorhexidine towels	140 (95.9)	94 (96.9)	46 (93.9)	NS ^‡^
Soap and water	6 (4.1)	3 (3.1)	3 (6.1)	
Were you allowed to wash your genitals by yourself?				
n (%)	85 (61.6)	44 (49.4)	41 (83.7)	<0.05
If the answer was NO, Would you have preferred to wash your genitals by yourself? n (%) (n=49)				
Absolutely not	22 (44.9)	22 (53.7)	0	0.036
A little	11 (22.4)	8 (19.5)	3 (37.5)	
Pretty much	11 (22.4)	7 (17.1)	4 (50)	
A lot	5 (10.2)	4 (9.8)	1 (12.5)	

p ^*^ = Significance level; chi-square test (p-values<0.05 were considered significant); ^†^SD = Standard Deviation. Student’s t test; ^‡^NS = Not Significant

40% of the patients from Group B felt that their intimacy was pretty much or a lot invaded and significantly, against 3% from Group A.

In both groups, the hygiene procedures were performed by a mean of approximately two professionals; however, 1 out of 4 patients from Group B would have preferred fewer people.

More than 70% of the patients like that the professionals talk to them, tell them stories and show professionalism while performing the hygiene procedures. However, those who had already been subjected to these procedures highlight it in a higher percentage.

It was significant that the patients from Group A assessed the cleanliness sensation more positively than those from Group B.

The Group B patients were offered to wash their genitals by themselves significantly more often; in addition, all the patients from this group preferred to perform this task on their own.

Table 3 shows technical aspects of the hygiene procedures such as interruptions, which were significantly higher in number in Group B, and the fact that the door was opened more frequently during the procedures. 83% of the Group B patients were a little upset by the interruptions, against 24% in Group A. In addition, 76% of the Group B patients were a little upset that the door was opened, against 32% in Group A. 

Most of the patients did not feel pain or were hot/cold during the hygiene procedures.

**Table 3 -:** Technical aspects of the hygiene procedures: differences based on previous experiences related to hygiene in bed. Barcelona, Spain, 2021

Technical aspects of the hygiene procedures	TOTAL (n=148)	Grupo A Hygiene in bed already performed (n=99)	Grupo B Hygiene in bed never performed (n=49)	p ^*^
Were there any interruptions during the hygiene procedures?				
n (%) YES	41 (27.7)	22 (22.2)	19 (38.8)	0.028
Did the interruptions upset you? n (%) (n=39)				
Absolutely not	19 (48.7)	16 (76.2)	3 (16.7)	0.002
A little	12 (30.8)	4 (19)	8 (44.4)	
Pretty much	6 (15.4)	1 (4.8)	5 (27.8)	
A lot	2 (5.1)	0	2 (11.1)	
Was the door kept closed?				
n (%) YES	98 (66.2)	73 (73.7)	25 (51)	0.005
Were you upset that they opened the door? n (%) (n=46)				
Absolutely not	22 (47.8)	17 (68)	5 (23.8)	0.005
A little	14 (30.4)	7 (28)	7 (33.3)	
Pretty much	8 (17.4)	1 (4)	7 (33.3)	
A lot	2 (4.3)	0	2 (9.5)	
Did you feel pain? n (%)				
Absolutely not	123 (83.7)	87 (87.9)	36 (75)	NS ^†^
A little	19 (12.9)	11 (11.1)	8 (16.7)	
Pretty much	4 (2.7)	1 (1)	3 (6.3)	
A lot	1 (0.7)	0	1 (2.1)	
Were you cold or hot? n (%)				
It was fine	101 (69.2)	69 (71.1)	32 (65.3)	NS ^†^
A little cold	41 (28.1)	24 (24.7)	17 (34.7)	
Very cold	4 (2.7)	4 (4.1)	0	
Hygiene shift n (%)				
Morning	90 (60.8)	57 (57.6)	33 (67.3)	NS ^†^
Night	56 (37.8)	41 (41.4)	15 (30.6)	
Afternoon	2 (1.4)	1 (1)	1 (2)	
Preferred shift for hygiene n (%)				
Morning	119 (80.4)	79 (79.8)	40 (81.6)	NS ^†^
Night	28 (18.9)	20 (20.2)	8 (16.3)	
Afternoon	1 (0.7)	0	1 (2)	
Did they wake you up for the hygiene procedures?				NS ^†^
n (%) YES	48 (32.9)	35 (35.7)	13 (27.1)	NS ^†^
Did you have to undergo any test/intervention in the following 3 hours?				
n (%) YES	42 (29.2)	30 (30.9)	12 (25.5)	NS ^†^

p ^*^ = Significance level; chi-square test (p-values<0.05 were considered significant); ^†^NS = Not Significant

In the CICU, 38% of the hygiene procedures performed are developed during the night shift, although they generally take place between 6 am and 8 am, and 87.5% of the patients had to undergo some test or surgical intervention in the following 3 hours.

In 67% of the cases, the hygiene procedures are in charge of two women, whereas 32% of the procedures are performed by a man and a woman. No differences were found between the genders. Table 4 shows the differences between both groups regarding the gender of the professionals performing the hygiene procedures and the patients’ preferences. 

**Table 4 -:** Gender of the professionals involved in the hygiene procedures and preferences: differences based on previous experiences related to hygiene in bed. Barcelona, Spain, 2021

Gender of the professionals involved in the hygiene procedures and preferences	TOTAL (n=148)	Grupo A Hygiene in bed already performed (n=99)	Grupo B Hygiene in bed never performed (n=49)	p ^*^
Who performed the hygiene procedures, by gender? n (%)				
Woman	99 (66.9)	63 (63.6)	36 (73.5)	NS ^†^
Woman/Man	48 (32.4)	36 (36.4)	12 (24.5)	
Man	1 (0.7)	0	1 (2)	
Who would you have preferred, by gender? n (%)				
Woman	110 (74.3)	73 (73.7)	37 (75.5)	NS ^†^
Woman/Man	31 (20.9)	21 (21.2)	10 (20.4)	
Man	7 (4.7)	5 (5.1)	2 (4.1)	
Would you prefer that a family member performed or collaborated in the hygiene procedures?				NS ^†^
n (%) SIM	47 (32)	34 (34.7)	13 (26.5)	
If the answer was YES, indicate kinship n (%) (N=48)				
Wife	22 (45.8)	19 (55.9)	3 (21.4)	<0.05
Partner	4 (8.3)	1 (2.9)	3 (21.4)	
Daughter	15 (31.3)	14 (41.2)	1 (7.1)	
Mother	6 (12.5)	0	6 (42.9)	
Others	1 (2.1)	0	1 (7.1)	

p ^*^ = Significance level; chi-square test (p-values<0.05 were considered significant); ^†^NS = Not Significant

32% of the patients would prefer that a family member performed or collaborated in the hygiene procedures, with no differences between the groups. In the open question, 5% of the patients stated preferring the hygiene procedures to be performed with soap and water rather than with chlorhexidine towels.

## Discussion

Health care should be individualized and patient-centered care must be implemented; it is for this reason that studies focused on the patients’ experience gain increasing relevance ^(^ 4 ^-^ 5 ^,^ 9 ^)^ . There is not much evidence about ICU Nursing care experiences, and it is mainly limited to qualitative studies ^(^ 13 ^,^ 25 ^)^ . 

As not everything can be individualized due to the occasional influence of ICU organization, personnel or infrastructure, it is necessary to know what most of the patients think, in order to adapt our units for the benefit of more patients.

Most of the conscious patients who had been subjected to hygiene procedures in bed for the first time are embarrassed, whereas 40% feel that their intimacy is pretty much or a lot invaded.

They are also mostly upset that the doors are opened or by interruptions. However, on later admissions, the patients no longer feel that the hygiene procedures in bed are an invasion to their intimacy: they state more conformity towards this technique and find contact with the Nursing personnel (while talking during the procedure) or their professionalism more pleasant, in addition to enjoying the cleanliness sensation more.

The patients that had never been subjected to hygiene procedures in bed are also younger and mostly single, which might intensify the fact that they are more embarrassed.

In 67% of the cases, the hygiene procedures are in charge of two women, whereas 32% of the procedures are performed by a man and a woman. This fact is comparable to the gender of the health professionals from the unit.

Interruptions are frequent in the ICU; 1 interruption every 3 minutes is estimated, mostly related to the work dynamics or to the patients ^(^ 3 ^,^ 26 ^)^ . From the results of our study, it is inferred that there were significantly more interruptions and that the doors were opened more times for the Group B patients. This fact turns out to be unexpected, and it might as well respond to these patients being more aware of such interruptions, which were more upsetting. 

Although the recommendation is to favor nocturnal rest and not performing any hygiene procedures during the night, one-third of these patients are subjected to the procedures during the night shift, generally from 6 am to 8 am, following the hospital’s organization system. 12.5% of these patients were woken up to perform this technique with no early test or intervention ahead ^(^ 24 ^)^ . We need to continue working in this sense to favor the patients’ rest whenever possible. 

Pain is the main stressor faced by patients in the ICU ^(^ 8 ^,^ 18 ^,^ 27 ^)^ . It is important to note that the patients under study assessed the hygiene procedures as not painful. 

The fact that, according to the bibliography ^(^ 18 ^)^ , being away from the family is one of the most stressful factors inherent to the ICU is in opposition to what was found in this study, which detected a reduced percentage of patients that preferred their family participating in their care. The patients under study were conscious and mostly stable when the survey was applied, which in addition was administered between 24 and 48 hours after admission, a fact that might exert an influence on them not wanting their family members to be involved in ICU care. 

Participation of the families can improve the psychosocial, emotional and physical results of critically-ill patients and mitigate the risk of psychological morbidity related to their ICU experience ^(^ 22 ^-^ 23 ^)^ . It is for this reason that it would be necessary to develop studies with patients that remain hospitalized in an ICU for extended periods of time, as longer ICU admissions generate more negative experiences ^(^ 15 ^,^ 18 ^)^ , as well as to analyze the family’s opinion about its implication in collaborating with the care provided. 

Cardiac patients are chronic and are usually readmitted due to decompensations or deterioration throughout their life. As health professionals, it is a good thing for us to think that the patients find the hygiene procedures more pleasant and enjoy our company more in their subsequent admissions.

A qualitative study from 2018 assessed the patients’ perception about hygiene procedures in ICUs, finding negative sensations such as disrespect and lack of sensitivity on the part of the professionals ^(^ 13 ^)^ . In our study, the patients’ perception was mostly positive, emphasizing that they liked having contact and communication with the Nursing personnel. Possibly, we have improved the care we provide to the patients in the last few years, placing them at the center of the assistance offered, which favors a better perception about their hospitalization in the ICU ^(^ 21 ^)^ . 

Talking to the patients while performing the hygiene procedures can contribute to minimizing stress in the ICU ^(^ 5 ^,^ 20 ^)^ . According to a study that evaluates the perception of hygiene with chlorhexidine towels, this practice is rejected by 16% of the patients. In our study, only 5% stated preferring hygiene with soap and water; this can be due to the fact that the patients are explained the expected benefit of infection prevention practices that are performed on a daily basis ^(^ 25 ^)^ . 

The study was conducted with visit restrictions due to the COVID-19 pandemic, limited to one hour in the morning and one in the afternoon and not allowing family member turnover, which precluded extended visit times. Along with the survey being applied during the first two days after admission, this fact may have exerted and influence on the patients not wanting their family member to be involved in the care provided in the CICU.

The data from 2018 were used for sample calculation since, for organizational reasons, there were more sedated and intubated patients during 2019, not representative of the unit’s trend. The data from 2020 were also discarded for sample calculation, as they underwent changes as a consequence of the COVID-19 pandemic.

As study limitations, the questionnaire was not validated by any analysis of its psychometric properties (reliability, validity, etc.) and the initial test was performed with only 5 subjects, which might imply certain preparation bias. The study design failed to contemplate the gender perspective and the patients were categorized according to their biological sex.

## Conclusion

This study concludes that, in general, the patients do not feel any invasion to their intimacy when they are subjected to hygiene procedures in bed and that they appreciate fluid communication with the health personnel while this care is developed.

The patients that are admitted to the CICU for the first time and who had never been subjected to hygiene procedures in bed are younger, feel more embarrassed with the health professionals and are more upset with the interruptions, in addition to being more aware of them.

As the professionals in charge of the hygiene of critically-ill patients, nurses should seek to preserve intimacy while the technique is performed and to minimize the interruptions that might upset the patients.

The patients value and appreciate being given the opportunity to wash their genitals by themselves. It is necessary to sensitize our professionals about the importance of actively involving them during the procedure, systematically inviting the patients to wash all the body areas they can (including the genitals) by themselves, whenever possible.

Some of the patients would appreciate that a family member took part in the hygiene procedures. Future studies are required to assess the family members’ willingness to more actively participate in the care provided.
